# Multi-omic studies on missense *PLG* variants in families with otitis media

**DOI:** 10.1038/s41598-020-70498-w

**Published:** 2020-09-14

**Authors:** Tori C. Bootpetch, Lena Hafrén, Christina L. Elling, Erin E. Baschal, Ani W. Manichaikul, Harold S. Pine, Wasyl Szeremeta, Melissa A. Scholes, Stephen P. Cass, Eric D. Larson, Kenny H. Chan, Rafaqat Ishaq, Jeremy D. Prager, Rehan S. Shaikh, Samuel P. Gubbels, Ayesha Yousaf, University of Washington Center for Mendelian Genomics (UW-CMG), Michael J. Bamshad, Deborah A. Nickerson, Suzanne M. Leal, Todd M. Wine, Michael J. Bamshad, Patricia J. Yoon, Herman A. Jenkins, Deborah A. Nickerson, Sven-Olrik Streubel, Norman R. Friedman, Daniel N. Frank, Elisabet Einarsdottir, Juha Kere, Saima Riazuddin, Kathleen A. Daly, Suzanne M. Leal, Allen F. Ryan, Petri S. Mattila, Zubair M. Ahmed, Michele M. Sale, Tasnee Chonmaitree, Regie Lyn P. Santos-Cortez

**Affiliations:** 1 Department of Otolaryngology-Head and Neck Surgery, School of Medicine, University of Colorado Anschutz Medical Campus, Aurora, CO USA; 2 Department of Otorhinolaryngology, Head and Neck Surgery, University of Helsinki and Helsinki University Hospital, Helsinki, Finland; 3 Human Medical Genetics and Genomics Program, University of Colorado Anschutz Medical Campus, Aurora, CO USA; 4 Center for Public Health Genomics, School of Medicine, University of Virginia, Charlottesville, VA USA; 5 Department of Otolaryngology, University of Texas Medical Branch, Galveston, TX USA; 6 Department of Pediatric Otolaryngology, Children’s Hospital Colorado, Aurora, CO USA; 7 Department of Otorhinolaryngology, Head and Neck Surgery, School of Medicine, University of Maryland, Baltimore, MD USA; 8 Institute of Molecular Biology and Biotechnology, Bahauddin Zakariya University, Multan, Punjab Pakistan; 9 Department of Genome Sciences, University of Washington, Seattle, WA USA; 10 Division of Infectious Diseases, Department of Medicine, School of Medicine, University of Colorado Anschutz Medical Campus, Aurora, CO USA; 11 Folkhälsan Institute of Genetics and Molecular Neurology Research Program, University of Helsinki, Helsinki, Finland; 12 Department of Biosciences and Nutrition, Karolinska Institutet, Huddinge, Sweden; 13 Department of Medical and Molecular Genetics, King’s College London, London, UK; 14 Department of Otolaryngology, Head and Neck Surgery, University of Minnesota, Minneapolis, MN USA; 15 Department of Neurology, Center for Statistical Genetics, Gertrude H. Sergievsky Center, Taub Institute for Alzheimer’s Disease and the Aging Brain, Columbia University Medical Center, New York, NY USA; 16 Division of Otolaryngology, Department of Surgery, UCSD School of Medicine and VA Medical Center, La Jolla, CA USA; 17 Department of Public Health Sciences, School of Medicine, University of Virginia, Charlottesville, VA USA; 18 Department of Biochemistry and Molecular Genetics, School of Medicine, University of Virginia, Charlottesville, VA USA; 19 Division of Infectious Diseases, Department of Pediatrics, University of Texas Medical Branch, Galveston, TX USA; 20 Center for Children’s Surgery, Children’s Hospital Colorado, Aurora, CO USA

**Keywords:** Medical genetics, Genetic predisposition to disease, Genetic association study, DNA sequencing, Next-generation sequencing, RNA sequencing, Sequence annotation, Targeted resequencing, Microbial genetics, Bacterial genetics, Genetics, Gene expression

## Abstract

Otitis media (OM), a very common disease in young children, can result in hearing loss. In order to potentially replicate previously reported associations between OM and *PLG,* exome and Sanger sequencing, RNA-sequencing of saliva and middle ear samples, 16S rRNA sequencing, molecular modeling, and statistical analyses including transmission disequilibrium tests (TDT) were performed in a multi-ethnic cohort of 718 families and simplex cases with OM. We identified four rare *PLG* variants c.112A > G (p.Lys38Glu), c.782G > A (p.Arg261His), c.1481C > T (p.Ala494Val) and c.2045 T > A (p.Ile682Asn), and one common variant c.1414G > A (p.Asp472Asn). However TDT analyses for these *PLG* variants did not demonstrate association with OM in 314 families. Additionally *PLG* expression is very low or absent in normal or diseased middle ear in mouse and human, and salivary expression and microbial α-diversity were non-significant in c.1414G > A (p.Asp472Asn) carriers. Based on molecular modeling, the novel rare variants particularly c.782G > A (p.Arg261His) and c.2045 T > A (p.Ile682Asn) were predicted to affect protein structure. Exploration of other potential disease mechanisms will help elucidate how *PLG* contributes to OM susceptibility in humans. Our results underline the importance of following up findings from genome-wide association through replication studies, preferably using multi-omic datasets.

## Introduction

Otitis media (OM) is one of the most common diseases in young children, with 80% of children under the age of 3 years having at least one episode of acute OM^1^. OM with effusion can result in hearing loss^2^, and can occasionally lead to an auditory processing disorder^3^. The frequent occurrence of this disease continues to be a burden on the healthcare system. From 2009 to 2011, 8.6 million emergency department visits resulted in otologic diagnoses in the United States, with OM as the most common diagnosis (60.6%) for ear cases^4^. This healthcare burden results in great costs. Management of OM, including office visits, antibiotics, and surgeries from 1998–2008 was estimated to have cost over $5.3 billion annually in the United States^5^.

There are multiple known risk factors for OM, such as allergies, viral upper respiratory infection, previous history of OM, family history, lack of breastfeeding, multiple siblings, day care attendance, second-hand smoke and low social status^6^. Despite the public health measures taken to lower these risk factors and reduce the incidence of OM, prevalence remains high, suggesting additional factors such as genetic predisposition. Previous studies have shown a strong correlation between genetic risk factors and both recurrent acute otitis media (RAOM) and chronic otitis media with effusion (COME), with heritability estimated from 22–74% based on type of OM and cohort^7,8^.

In a genome-wide association study (GWAS) involving > 120,000 European-descent individuals, a heterozygous *PLG* variant c.112A > G (p.Lys38Glu) was associated with childhood ear infections [OR = 1.43; 95%CI = 1.26, 1.63; *p* = 3.8 × 10^−8^] ^9^. Additionally *PLG* variants c.112A > G (p.Lys38Glu) and c.1414G > A (p.Asp472Asn) were reported to be co-inherited in families with type I plasminogen deficiency^10^.

*PLG* (MIM 173,350) encodes plasminogen which is a secreted blood zymogen that, when activated by proteolysis, is converted to plasmin. The product plasmin is a serine protease that degrades fibrin clots and promotes degradation of the extracellular matrix^11^. Plasminogen has been shown to play a crucial role in other cellular processes such as wound healing, immunity, tissue remodeling, inflammation, and cell migration^12^. Interestingly, there is evidence that some bacteria possess plasminogen-binding adhesins on their cell surface to exploit the fibrinolytic system by degrading host junction proteins and initiating signaling events that facilitate bacterial uptake and invasion^11,13^. In previous studies, mice deficient in plasminogen spontaneously developed chronic OM by 18 weeks of age, and local injection of plasminogen led to healing of eardrum perforations^14,15^. For these mouse studies, only homozygous *Plg*-deficient mice were examined.

In this study, we want to determine whether individuals with heterozygous *PLG* variants also have increased susceptibility to OM. We aim to identify coding *PLG* variants co-segregating in families with OM. For the identified *PLG* variants, we followed up our findings with phenotypic description, tissue expression and microbiome analyses to determine the role that these variants potentially play in OM pathogenesis. Identification of associations between pathogenic variants and OM will increase the overall understanding of OM susceptibility and the mechanisms behind the disease.

## Results

### PLG variants identified from exome data in multi-ethnic families with OM

**Figure 1:**
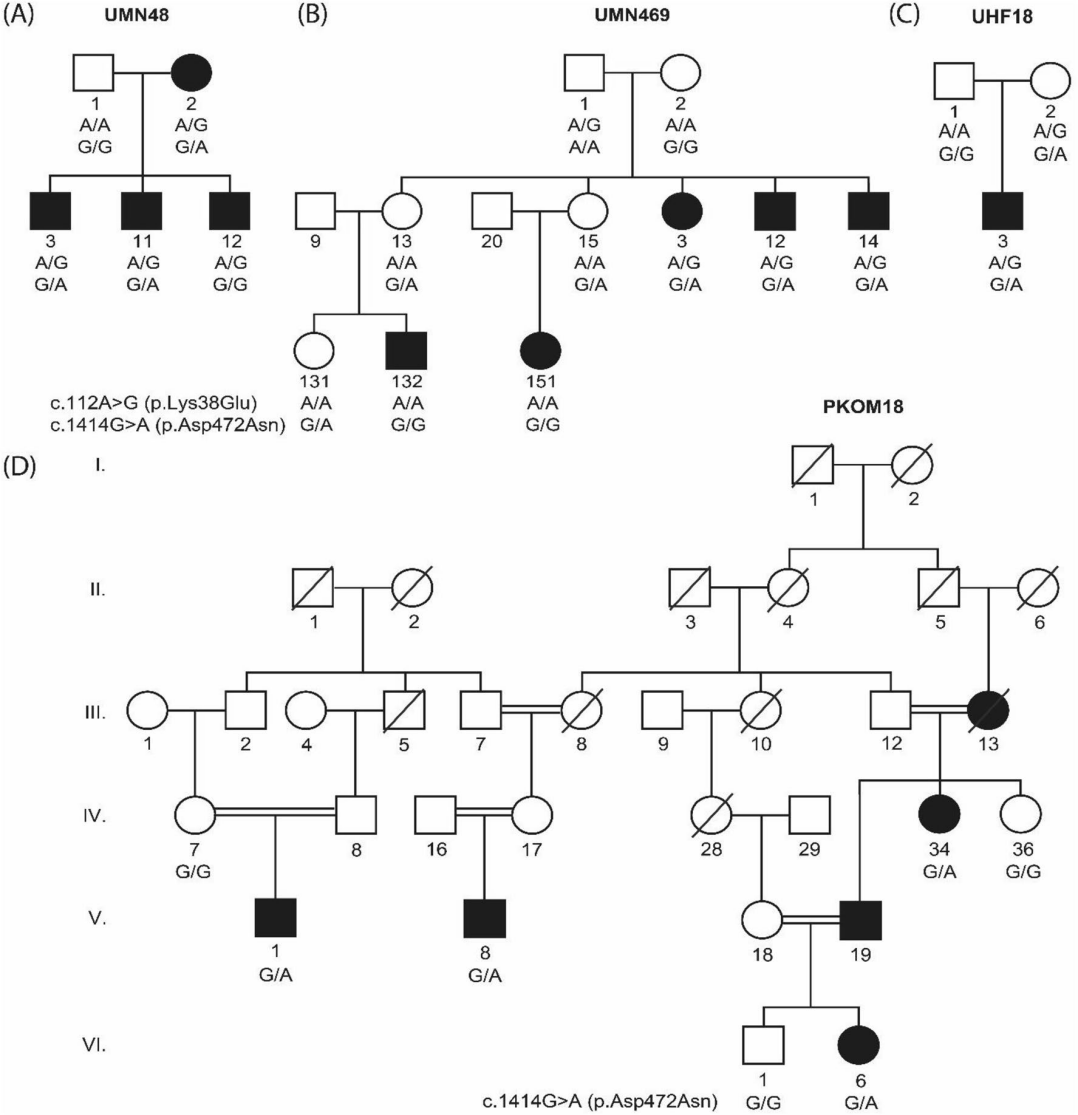
Four multi-ethnic families with the *PLG* c.112A > G (p.Lys38Glu) and c.1414G > A (p.Asp472Asn) variants. Two Minnesota families (**A**) UMN48 and (**B**) UMN469 and (**C**) one Finnish trio UHF18 carry both variants with an autosomal dominant pattern of inheritance with reduced penetrance. (**D**) Despite Pakistani family PKOM18 being highly consanguineous, *PLG* c.1414G > A (p.Asp472Asn) co-segregated with OM in an autosomal dominant pattern.

**Figure 2:**
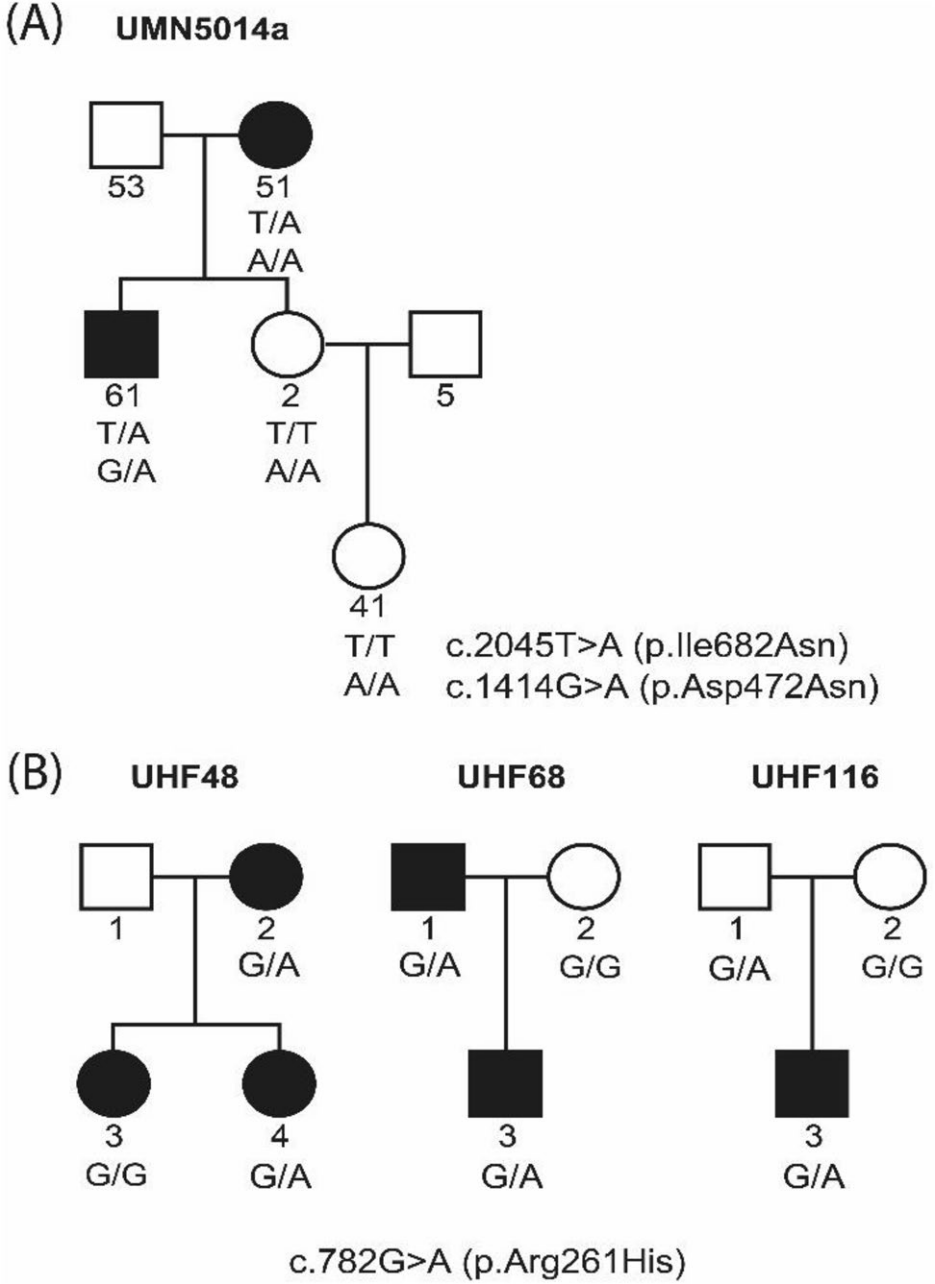
Four multi-ethnic families with the *PLG* c.782G > A (p.Arg261His), c.2045 T > A (p.Ile682Asn) and c.1414G > A (p.Asp472Asn) variants. (**A**) In Minnesota family UMN5014a, the c.2045 T > A (p.Ile682Asn) variant co-segregates with OM, however two unaffected individuals carry the c.1414G > A (p.Asp472Asn) variant. (**B**) Three Finnish families have the c.782G > A (p.Arg261His) variant.

We submitted DNA samples from 259 multi-ethnic families for exome sequencing. In nine families, we identified four rare and damaging *PLG* variants c.112A > G (p.Lys38Glu), c.782G > A (p.Arg261His), c.1481C > T (p.Ala494Val) and c.2045 T > A (p.Ile682Asn), and one common and benign variant c.1414G > A (p.Asp472Asn; Table 1; Figs. 1; 2). The variants c.112A > G (p.Lys38Glu) and c.1414G > A (p.Asp472Asn) were both found in two Minnesota families and one Finnish trio with an autosomal dominant pattern of inheritance with reduced penetrance (Fig. 1A–C). For each identified exome variant, Sanger sequencing of the same variant(s) was performed in the rest of the relatives of the proband to check for co-segregation. Family UMN48 co-segregates c.112A > G (p.Lys38Glu) with OM, however only two out of three affected siblings are heterozygous for c.1414G > A (p.Asp472Asn) due to a recombination event between these two variants in the third sibling (Fig. 1A). The second Minnesota family UMN469 has incomplete co-segregation for both c.112A > G (p.Lys38Glu) and c.1414G > A (p.Asp472Asn); only the affected individuals in the second generation are heterozygous for both variants, while the affected children in the third generation are wildtype, which may suggest intra-familial genetic heterogeneity (Fig. 1B) ^16,17^. Of the seven family members of UMN469 that carry the c.1414G > A (p.Asp472Asn) variant, four are unaffected with OM (Fig. 1B). The third family is Finnish trio UHF18: the proband has both c.112A > G (p.Lys38Glu) and c.1414G > A (p.Asp472Asn), but the mother who also has both c.112A > G (p.Lys38Glu) and c.1414G > A (p.Asp472Asn) was normal at examination and has no written record of being affected with OM in childhood (Fig. 1C).

**Table 1:** *PLG* variants identified in nine multi-ethnic families with otitis media.

ID	hg19 Position^1^	cDNA variant	Amino acid variant	rsID	Damaging prediction^2^	CADD	gnomAD MAF
UMN48^3^, UMN469^3^, UHF18^3^	161,127,501	c.112A > G	p.Lys38Glu	rs73015965	FA,MA,mLR,mSVM, MT,PP2,SI	19.0	NFE = 0.005, FIN = 0.0005
UHF48, UHF68, UHF116	161,137,790	c.782G > A	p.Arg261His	rs4252187	MA,MT,PP2,PR,SI	27.6	NFE = 0.004, FIN = 0.005
UMN48^3^, UMN469^3^, PKOM18, UHF18^3^, UMN5014a^4^	161,152,240	c.1414G > A	p.Asp472Asn	rs4252125	–	1.4	NFE = 0.29, SAS = 0.10, FIN = 0.26
UHF520	161,152,819	c.1481C > T	p.Ala494Val	rs4252128	MA, SI, PP2	26.8	NFE = 0.004, FIN = 0.00004
UMN5014a^4^	161,162,369	c.2045 T > A	p.Ile682Asn	rs147175166	FA,MT,PP2,PR	23.3	NFE = 0.001

*CADD* scaled combined annotation-dependent depletion score, *FA* FATHMM, *FIN* gnomAD finnish, *gnomAD* genome aggregation database, *MA* mutation assessor, *MAF* population-matched minor allele frequency, *mLR* MetaLR, *mSVM* MetaSVM, *MT* MutationTaster, *NFE* gnomAD non-Finnish European, *PKOM* Pakistani family; *PP2* PolyPhen2 HVAR, *PR* PROVEAN, *SAS* gnomAD South Asian, *SI* SIFT, *UHF* Finnish family, *UMN* Minnesota family.

^1^mRNA accession number = NM_00301.

^2^Damaging prediction from bioinformatics tools in dbNSFP v.3.3a.

^3^Families UMN48, UMN469, and UHF18 have both the c.112A > G (p.Lys38Glu) and the c.1414G > A (p.Asp472Asn) variant. In two families UMN469 and UHF48, not all affected individuals carry the *PLG* variant. In one trio UHF18, one unaffected individual carries both variants.

^4^Family UMN5014a has both the c.1414G > A (p.Asp472Asn) and the c.2045 T > A (p.Ile682Asn) variant. Two unaffected individuals carry the c.1414G > A (p.Asp472Asn) variant.

The exome data from an affected individual from Pakistani family PKOM18 is heterozygous for c.1414G > A (p.Asp472Asn) but this individual does not carry the c.112A > G (p.Lys38Glu) variant. Despite PKOM18 being highly consanguineous, Sanger sequencing of the DNA samples from the rest of the PKOM18 family showed that the c.1414G > A (p.Asp472Asn) variant co-segregated with OM in an autosomal dominant pattern (Fig. 1D).

Additional rare, predicted-to-be-damaging *PLG* variants co-segregated with OM in four families (Fig. 2; Table 1). In one Minnesota family UMN5014a, the c.2045 T > A (p.Ile682Asn) variant co-segregates with OM. However, in this family all relatives with DNA samples including two affected and two unaffected individuals carry the c.1414G > A (p.Asp472Asn) variant (Fig. 2A). Three Finnish families have the c.782G > A (p.Arg261His) variant (Fig. 2B). Two out of three affected individuals in family UHF48 carry the c.782G > A (p.Arg261His) variant. In two Finnish trios, UHF68 and UHF116, the proband is heterozygous for the c.782G > A (p.Arg261His) variant. In both trios the variant was inherited from the father, but the father was considered affected in only one of these two trios (Fig. 2B). We identified c.1481C > T (p.Ala494Val) in the exome data for one Finnish individual with OM, however DNA was not available for both parents, and therefore we could not verify co-segregation or inheritance within that family (pedigree not shown).

In summary, three out of five families with multiple affected relatives co-segregate at least one *PLG* variant with OM. In three trios the *PLG* variant was inherited, but in only one trio was a parent who transmitted the minor allele for the *PLG* variant labeled as affected. One affected proband without family data carried the minor allele for a rare *PLG* variant.

In the five Finnish families with *PLG* variants and exome data, five out of the eight affected individuals have both RAOM and COME. We also checked an additional 25 Minnesota and 213 Finnish families with exome data for additional *PLG* variants, and only found the common c.1414G > A (p.Asp472Asn) variant in 16 (64%) Minnesota and 95 (45%) Finnish families. However the allele counts in these families are not significantly different from counts for the c.1414G > A (p.Asp472Asn) variant in non-Finnish European and Finnish gnomAD alleles. In Finnish families, no significant association was found between the *PLG* c.1414G > A (p.Asp472Asn) variant and OM type. Additionally, in OM patients from Colorado and Texas, carriage of either c.112A > G (p.Lys38Glu) or c.1414G > A (p.Asp472Asn) was not associated with either RAOM or COME. These data suggest that *PLG* variants are not associated with a specific OM type.

### Additional non-OM traits in families with PLG variants

In three Minnesota families with heterozygous *PLG* variants, two of which have [c.112A > G(p.Lys38Glu);c.1414G > A(p.Asp472Asn)] and one with [c.2045 T > A(p.Ile682Asn);c.1414G > A(p.Asp472Asn)], a higher proportion of affected individuals have hay fever, asthma or allergy, with allergy being significant (Fisher exact *p* = 0.047). On the other hand, out of five Finnish families with *PLG* variants, in family UHF48 only one of two affected individuals had asthma, as well as hypertension and psoriasis. A second family UHF18 had other potentially autoimmune phenotypes such as diabetes, nephritis and uveitis. In Pakistani family PKOM18, only one affected relative had eczema. In three Texan trios with transmission of the minor allele for the c.112A > G (p.Lys38Glu) variant (Table 2), only one out of six variant carriers have allergic rhinitis history. Taken together, these findings do not show a consistent pattern of association across cohorts between heterozygous *PLG* variants and additional traits.

**Table 2:** Transmission disequilibrium test for *PLG* variants in 314 US trios.

Variant	White	Hispanic	Black	Mixed/Other	Transmitted	Non-transmitted	TDT *p*
p.Lys38Glu	232	44	21	17	3	4	0.71
p.Arg261His	30	3	0	7	0	0	–
p.Ala494Val	49	7	2	9	0	1	0.31
p.Ile682Asn	190	41	22	10	1	0	0.32
p.Asp472Asn	221	44	23	17	101	116	0.31
All variants^1^	215	43	21	17	104	119	0.32

^1^For UTMB59, the father transmitted the minor allele for both c.112A > G (p.Lys38Glu) and c.1414G > A (p.Asp472Asn) variants to the child. On the other hand, for each of two trios UTMB132 and UTMB206 one parent carried both c.112A > G (p.Lys38Glu) and c.1414G > A (p.Asp472Asn) variants but did not transmit the minor alleles to the child.

### TDT analysis for PLG variants in US trios

In order to potentially replicate OM GWAS findings for *PLG,* the c.112A > G (p.Lys38Glu), c.782G > A (p.Arg261His), c.1481C > T (p.Ala494Val), c.2045 T > A (p.Ile682Asn), and c.1414G > A (p.Asp472Asn) variants were Sanger-sequenced in 314 US trios with OM. To determine genetic linkage and association, transmission of the minor alleles for these *PLG* variants were assessed using the transmission disequilibrium test (TDT)^18^. The minor allele for variant c.782G > A (p.Arg261His) was not present in Colorado trios. The minor allele for c.1481C > T (p.Ala494Val) was non-transmitted in one trio and transmitted in no trios (Table 2). For c.112A > G (p.Lys38Glu), three alleles were transmitted and four were non-transmitted. The variant c.1414G > A (p.Asp472Asn) also had a higher number of non-transmitted alleles, with 101 transmitted and 116 non-transmitted alleles. Lastly, the variant c.2045 T > A (p.Ile682Asn) had one transmitted allele and zero non-transmitted. Based on the TDT *p*-values for all variants, and their combined *p* value (*p* = 0.32), these variants were not significant in family-based association tests. Based on posthoc power analysis^19^, and an odds ratio of 1.43 for the *PLG* variant c.112A > G (p.Lys38Glu) from GWAS^9^,our trio study is underpowered to detect this small effect size and will require at least 16,200 trios to obtain sufficient power. On the other hand, in a previous study we were able to obtain a significant result for a rare *FUT2* c.412C > T (p.Arg138Cys) variant using the same trio set^16^.

### Low or absent PLG expression in saliva and middle ear

*Plg* expression was checked in microarray data of wildtype mouse middle ears that were inoculated with non-typeable *Haemophilus influenzae* (NTHi) vs. control^20^. *Plg* expression was low in mouse middle ear. In general, regulation of *Plg* in the mouse middle ear following infection with NTHi was minimal, although significant down-regulation was observed at a single time point at 48 h after inoculation.

From human data from GTEx, *PLG* is most highly expressed in the liver, with lower expression in the kidney and spleen. However no GTEx data is available on middle ear expression and the four rare *PLG* variants we identified. On the other hand, in GTEx, the common c.1414G > A (p.Asp472Asn) variant is a significant expression quantitative trait locus (eQTL) in esophageal mucosa. We previously performed RNA-sequencing of 19 saliva samples from OM patients in the US^21^. In these saliva samples, *PLG* was expressed at a very low level (DESeq2 normalized reads: range 0 to 8.5). A Wilcoxon rank sum test was then used to compare the salivary *PLG* expression for individuals who are wildtype vs. carriers of c.1414G > A (p.Asp472Asn) variant alleles. There was no significant difference in *PLG* expression in saliva in the two groups (data not shown). Likewise *PLG* was expressed at a very low level in the cholesteatoma and mucosa samples collected during OM surgery (DESeq2 normalized reads: range 0 to 8.3, average cholesteatoma 1.96, average mucosa 2.79). Thus in our available samples from human saliva and middle ear, *PLG* expression was very low or absent.

### Network analysis

The *PLG* variant c.112A > G (p.Lys38Glu) was identified along with 31 other significant loci in a previous GWAS of OM^9,22^. In order to identify common biological processes between *PLG* and other loci associated with OM, this list of 32 genes was uploaded into NetworkAnalyst 3.0 and mapped (Reactome; Supplementary Figure S3A)^23–25^. In the resulting network, *SP3* and *PLG* were the two largest nodes and were joined by 4 different nodes: *SERPINE1*, *FN1*, *IGFBP3*, and *EP300*. A secondary network was then mapped from the gene list: *SP3*, *PLG*, *SERPINE1*, *FN1*, *IGFBP3*, and *EP300* (GO:BP; Supplementary Figure S3B). The most significant pathways identified within these networks from the Reactome database were “dissolution of fibrin clot”, “platelet degranulation” and “response to elevated platelet cytosolic Ca^2+^” (Supplementary Table S3), which are related to the known functions of plasminogen in fibrinolysis.

### 16S rRNA sequencing and analyses of middle ear and nasopharyngeal samples from OM patients in the US according to carriage of the c.1414G > A (p.Asp472Asn) variant

We also performed microbiome sequencing and analyses of the middle ears and nasopharynges of carriers of the c.1414G > A (p.Asp472Asn) variant compared to wildtype individuals, however we found no significant differences in overall microbiome composition based on α-diversity indices (Supplementary Table 1). Only Bifidobacterium had an increased relative abundance in the nasopharynx of variant carriers (nominal *p* = 0.0024), which was non-significant after correction for multiple testing (false discovery rate/FDR-adjusted *p* = 0.13; Supplementary Fig. 1). Unfortunately, we do not have saliva, middle ear or microbial samples for probands with the rare *PLG* variants.

### Predicted effect of three rare variants on PLG protein

All three novel variants [c.782G > A (p.Arg261His), c.1481C > T (p.Ala494Val) and c.2045 T > A (p.Ile682Asn)] are located near disulfide bonds between cysteine residues (Cys166-Cys243, Cys483-Cys524, and Cys548-Cys666 respectively) that are potentially important for protein stabilization^26^. More specifically, these disulfide bonds are important for the structure and interface between domains. If these bonds are impacted by these variants, the stability and enzymatic activity of the whole protein could be affected^27,28^.

The c.2045 T > A (p.Ile682Asn) variant is within the β-chain of the trypsin domain near the “94-shunt” surface loop which is an important component in the accessibility of the catalytic triad residues that are necessary for trypsin-like protease activity^29^. The 6-residue difference between the typical “99-loop” of serine proteases and the “94-shunt” in PLG is predicted to be an important factor in its increased range of specificity^30^. In the native form, the isoleucine at this position forms an H-bond with Pro593 (Supplementary Fig. 2A) whereas the asparagine variant is predicted to form two extra H-bonds with Cys567 and Pro683 (Supplementary Fig. 1B). Due to this variant, there is a potential decrease in the accessibility of the catalytic triad that mediates the main enzymatic function of the protein.

The c.782G > A (p.Arg261His) variant occurs within the kringle 2 (KR2) domain of the PLG α-chain and could disrupt the single disulfide bond that serves as the interface between the KR2 and KR3 domains. The closed conformation of the native PLG, where fibrin is bound and less active, is in part stabilized by a chloride ion bond located near Arg261^26,31^. The histidine variant is predicted to interfere with this chloride ion bond and destabilize the protein (Supplementary Fig. 2C–D). Additionally other previously identified variants within this region have been predicted to interfere with the interface between the serine protease domain and KR2^32^.

The c.1481C > T (p.Ala494Val) variant occurs within the KR5 domain of the β-chain^26^. The KR5 domain is crucial for the change in conformation from closed to open via fibrin binding to the lysine binding sites present in this domain^31^. There were no changes in the H-bonds observed for this variant compared to the native protein and based on the location of the valine variant, the effect of the extra side chain on the valine is predicted to be minimal (Supplementary Fig. 2E–F).

## Discussion

We identified four rare variants and one common variant within *PLG* in probands with OM. The *PLG* variants c.112A > G (p.Lys38Glu) or c.1414G > A (p.Asp472Asn) co-segregate with OM in two out of five families with multiple affected relatives (Fig. 1; Fig. 2A), but we do not have additional evidence from trio or expression studies to support association between these variants and OM. In a previous GWAS, the heterozygous genotype for c.112A > G (p.Lys38Glu) was a risk factor for OM. Additionally this variant was often found to be co-inherited *in cis* with c.1414G > A (p.Asp472Asn), which was previously associated with increased susceptibility to fungal infection in mice^33^. However we did not see an association between any of the five identified *PLG* variants and OM in our trios (Table 2). It might be that these variants have weak effects that are only detectable in very large cohorts, e.g. with an OR of 1.43 for the p.Lys38Glu variant, we would need > 16,000 trios to detect a risk effect^19^. Furthermore, our families with OM may have other common or rare variants in other genes that have stronger phenotypic effects, whether risk or protective variants, and are concealing the effect of these *PLG* variants.

Hereditary plasminogen deficiency with reduced protein activity is referred to as Type 1 Plasminogen Deficiency (MIM 217090). This disease is inherited in an autosomal recessive manner, and most commonly manifests as ligneous conjunctivitis, in which the wood-like pseudomembranous lesions develop on the mucous membranes in the eyes^34,35^. Furthermore, similar lesions have been observed on the skin and other mucous membranes including the middle ear, gingiva, respiratory tract, and gastrointestinal or female genital tract, with OM occurring in 14% of patients with plasminogen deficiency. Notably none of the *PLG* carriers among our families have these lesions. *PLG* c.112A > G (p.Lys38Glu) is the most commonly identified variant in 34% of patients with type 1 plasminogen deficiency, whether as homozygous or compound heterozygous^12^. However individuals with either homozygous or compound heterozygous genotypes for c.112A > G (p.Lys38Glu) may have decreased serum plasminogen activity and may also be healthy^10^. We also considered the possibility that the known *PLG* variants are only causal of susceptibility when inherited in an autosomal recessive manner. However in the few individuals in the Colorado cohort that are homozygous for c.1414G > A (p.Asp472Asn), we did not see a difference in salivary *PLG* expression or α-diversity in microbial samples compared to wildtype or heterozygous (Supplementary Table 1). Likewise, none of the affected individuals in our families appear to be compound heterozygous for *PLG* variants.

Plasminogen has a potential role in pathogenic invasion by bacteria that bind it and thus can activate fibrin degradation to aid entry via cell junctions^11^. It is not uncommon for pathogens to use the host plasminogen activation system as a means of invasion and many bacteria with PLG receptors have been identified, including *Haemophilus influenzae* and *Streptococcus pneumoniae*^36,37^. *Bifidobacterium* is a common probiotic for use in preventing recurrent acute OM though its efficacy is unproven^38^. Additionally, a previous study did not detect *Bifidobacterium* as having a significant role in acute OM, suggesting it may be a commensal colonizer of the nasopharynx^39^. Thus the increased relative abundance of *Bifidobacterium* in the c.1414G > A (p.Asp472Asn) variant as compared to the wild-type does not corroborate previous findings that this common variant is associated with reduced risk of OM.

In human and mouse middle ear tissue *PLG* expression was low. Additionally, plasminogen activator activity could not be detected in middle ear effusion samples from adult patients^40^. However, in the mouse middle ear study reported here, expression of the plasminogen activator gene (*Plat*) is upregulated in the middle ear six hours after NTHi inoculation, suggesting some Plg activity within middle ear. Plasminogen is a blood protein that is essential for wound healing, including healing of tympanic membrane perforations^41^. This is further supported by our network analyses (Supplementary Table 3), wherein the most significant pathways linking *PLG* to other OM susceptibility genes involve processes for coagulation and cell adhesion which are both important for healing of injured tissues. In plasminogen-deficient patients, ligneous lesions primarily develop on the tympanic membrane, rather than the middle ear mucosa or cavity^42^. Unfortunately, we do not have data from the tympanic membrane. There could be other proteins such as activators or binding partners involved that result in *PLG* having an indirect effect on OM susceptibility. For example, in the GTEx database the common c.1414G > A (p.Asp472Asn) variant is an eQTL for a lincRNA in esophageal mucosa, suggesting other potential disease mechanisms for *PLG* variants such as epigenetic regulation. These are however outside the scope of this study. Because PLG is secreted by the cell, it is possible that the protein is active in circulation and in the middle ear space, as shown by previous studies involving mice and secretion assays for other rare variants^12,14^.

Interestingly, in COS-7 cells transfected with PLG mutants, secretion kinetics of both the c.112A > G (p.Lys38Glu) and c.1414G > A (p.Asp472Asn) variants were similar to the wild-type control^12^. This further supports the finding that the effects of these two variants on OM susceptibility are very weak to none. On the other hand, in vitro transfection of COS-7 cells from other variants including missense substitutions of arginine resulted in decreased cell secretion of PLG, suggesting that other novel variants are potentially functional^12^.

Three rare, heterozygous *PLG* variants, c.782G > A (p.Arg261His), c.1481C > T (p.Ala494Val) and c.2045T > A (p.Ile682Asn), were identified in US and Finnish families with OM. Two of these variants are predicted to change conformation of the protein and either affect enzymatic function or ligand binding (Supplementary Fig. 2). A previously reported variant c.782G > A (p.Arg261His) was identified in both cases and controls with multiple sclerosis^43^, but is novel for OM. A variant at the same amino acid position p.Arg261Cys was identified in a family with all heterozygous individuals having reduced PLG levels to 64–68%^44^. This might suggest that c.782G > A (p.Arg261His), and other rare variants in *PLG,* are likely to be functional or pathogenic. Additionally, previously reported *PLG* variants that affect secretion kinetics in COS-7 cells fall within the same domains as the novel *PLG* variants reported here^12^: variant c.2045 T > A (p.Ile682Asn) occurs within the β-chain of the trypsin domain, c.1481C > T (p.Ala494Val) within the KR5 domain of the β-chain, and c.782G > A (p.Arg261His) fall within the kringle 2 (KR2) domain of the α-chain^45^. All three domains in which these identified variants occur harbor previously reported variants that affect secretion of PLG. Due to the rarity of these three novel variants, their effects on tissue expression or the microbiome cannot be assessed at present.

To conclude, we identified three novel rare, missense variants and two known *PLG* variants which co-segregate with OM in some, but not all of our families with these variants. We hypothesized that these variants may play a role in OM susceptibility, however there was no association between these *PLG* variants and OM in our trios. Although the novel rare variants we identified might have potential effects on protein structure based on molecular modeling, *PLG* expression is very low or absent in normal or diseased middle ear tissues in both mouse and human. Our previous studies support a common mechanism by which the OM susceptibility variant affects expression of the encoded protein and other genes downstream, which in turn shifts the middle ear microbiome by changing the overall biodiversity or relative abundance of specific pathogens or commensal taxa^16,21,46,47^. The mostly negative results of our multiple studies showed that this disease mechanism does not apply to *PLG* variants in relation to OM susceptibility in humans. Based on the *Plg-*knockout mouse model with chronic OM and non-healing eardrum perforations, *PLG* variants may act on the healing process within the tympanic membrane or the secretion of plasminogen into the middle ear cavity through other disease mechanisms, including epigenetic, that are not detectable by the methods used in this study. However such effects are easily concealed by other stronger factors contributing to OM susceptibility within the middle ear mucosa, including other risk or protective variants with larger phenotypic effects in our families. Our results underline the importance of following up findings from genome-wide association through replication studies, preferably using multi-omic datasets.

## Materials and methods

### Ethics approval

Guidelines from the Declaration of Helsinki were followed throughout the conduct of this study. Prior to start of the study, ethical approval was obtained from the following institutional review boards (IRB): Bahauddin Zakariya University; Colorado Multiple IRB; Helsinki University Hospital; University of Maryland Baltimore; University of Minnesota; University of Texas Medical Branch (UTMB) Galveston; University of Virginia; and University of Washington. Informed consent was obtained from all adult participants and parents of children enrolled in the study. In addition, approval was obtained from the Institutional Animal Care and Use Committee of the Veterans Affairs Medical Center, San Diego, California. All experiments were performed in accordance with the Animal Welfare Act and the Health Research Extension Act of 1985.

### Subject ascertainment

Except in Pakistan, OM-affected families from different cohorts were first identified upon referral of a child or proband for OM surgery (Supplementary Table 2). Samples were obtained from previously established family cohorts for OM, including 257 trios from Texas, 85 trios from Colorado, 140 families from Minnesota, 217 families from Finland, and 19 families from Pakistan. For Texan and Coloradan trios, clinical data from the proband including age, sex, breastfeeding history, allergic history, OM status and surgical technique, and saliva samples from both proband and parents were obtained. All saliva samples were collected using Oragene DNA collection kits and DNA was isolated using the manufacturer’s protocol.

In the three cohorts from Minnesota, Finland and Pakistan, DNA was extracted from blood samples provided by family members who participated in the study. These families were characterized as previously described^16,48^. In Minnesota, all family members were examined by an otolaryngologist and tested by tympanometry for middle ear function, and family members were considered affected if ≥ 2 data sources, whether otoscopy, tympanometry, medical records, or personal history, were positive for OM. For Finnish families, clinical data, including history of OM, risk factors for OM, and otolaryngologic surgery, were obtained. Finnish individuals were considered positive for OM if they had insertion of tympanostomy tubes, effusive OM for > 2 months, or recurrent OM (i.e., > 3 episodes in 6 months or > 4 episodes in 12 months). Families from Pakistan were identified through referral for familial occurrence of OM. For Pakistani families, age at onset and recurrence of OM episodes were determined from medical history and OM status by otoscopy. For all cohorts, individuals with known genetic, craniofacial, and immunodeficiency syndromes were excluded.

### DNA sequencing

DNA samples from individuals with OM from 28 Minnesota, 217 Finnish, and 14 Pakistani families were submitted for exome sequencing using an Illumina HiSeq instrument, at an average 40–60 × coverage. For the Minnesota and Finnish families, sequence capture was performed at the University of Washington using the Roche NimbleGen SeqCap EZ Human Exome Library v.2.0. For the Pakistani families, exome sequencing was performed at the University of Maryland and genomic libraries were recovered for exome enrichment using the Agilent SureSelect Human Expanded All Exon V5 (62 Mb) kit. For all exome data, alignment and variant calling were performed using Burrows-Wheeler Aligner^49^and Genome Analysis Toolkit^50^, respectively.

Sanger sequencing was used to confirm co-segregation of five *PLG* variants (NM_00301.3) identified in exome data from nine multi-ethnic families (Table 1). An additional 70 Coloradan trios, 246 Texan trios, 1 Finnish family, 2 Minnesota families, and 1 Pakistani family without exome data were Sanger-sequenced for the *PLG* variants.

### Bioinformatic and statistical analyses

*PLG* variants found in exome data from OM-affected individuals were selected based on whether they are predicted to be damaging according to bioinformatic tools, including the scaled Combined Annotation-Dependent Depletion (CADD) score ≥ 15.0^51^, FATHMM^52^, MutationAssessor^53^, MutationTaster^54^, PolyPhen2^55^, PROVEAN^56^, SIFT^57^, MetaLR and MetaSVM from dbNSFP^58,59^. Variants predicted to be damaging were further filtered for rare minor allele frequency (MAF < 1%), with the exception of common variant c.1414G > A (p.Asp472Asn), which was previously found to be co-inherited with c.112A > G (p.Lys38Glu). A Transmission Disequilibrium Test (TDT) was performed using genotypes for *PLG* variants c.782G > A (p.Arg261His) and c.1481C > T (p.Ala494Val) for Coloradan trios, and c.112A > G (p.Lys38Glu), c.1414G > A (p.Asp472Asn) and c.2045 T > A (p.Ile682Asn) for Coloradan and Texan trios^18^.

For 314 families with *PLG* variants, non-genetic variables such as age, sex, ethnicity, breastfeeding history, previous diagnoses of allergy or asthma, and type of OM were investigated. Logistic regression analyses were performed with OM type (RAOM, COME) as outcome variable, carriage of *PLG* variants as independent variable and other non-genetic variables as covariates. For Finnish probands, Fisher exact test was used to compare occurrence of RAOM vs. COME according to the *PLG* c.1414G > A (p.Asp472Asn) genotype. All variants tested were in Hardy–Weinberg equilibrium in the Texan, Coloradan and Finnish probands.

### RNA expression in infected mouse middle ear

*PLG* expression in the mouse middle ear was derived from microarray analysis of mRNA from wild-type mice with acute OM after inoculation with non-typeable H. influenzae (NTHi) compared to healthy control mice, as described previously^20^. In order to discover genes that are regulated during acute OM, transbullar inoculation of the middle ears were performed on 320 WBxB6 F1 hybrid mice with PBS (sham control) or NTHi. Two independent samples were generated for each time point after infection or PBS injection: 0 h (0 h, uninfected controls), 3 h, 6 h, 1 day (1d), 2d, 3d, 5d, and 7d, from initiation of OM to resolution. RNA was profiled on Affymetrix mouse 430 2.0 whole-genome microarrays. A total of 3,605 genes, approximately 14.4% of the mouse genome, defined the signature of acute, NTHi-induced OM across time.

### RNA expression in human saliva and middle ear

Salivary RNA samples were collected from 19 pediatric patients undergoing OM surgery at the Children’s Hospital Colorado (CHCO) and submitted for RNA sequencing, as previously described^21^. In brief, RNA samples were processed with the NuGen Trio RNA-Seq Kit (Tecan, Redwood City, CA, USA) at the University of Colorado Denver Genomics and Microarray Core and sequenced on an Illumina HiSeq 4,000 with average ~ 31 million reads per sample. Reads were trimmed using the FASTX-Toolkit (v0.0.13) and aligned using STAR v2.5.3a^60^. Counts were normalized, summed and analyzed according to carriage of *PLG* variant c.1414G > A (p.Asp472Asn) using the Wilcoxon test.

Three cholesteatoma samples and four mucosa samples were collected from patients undergoing OM surgery at the University of Colorado Hospital or CHCO. Samples were immediately isolated using the QIAGEN RNeasy Micro Kit. The tissue was initially homogenized with glass beads and QIAGEN buffer RLT, using a bead vortexer for 4 min. All four mucosa and three cholesteatoma samples passed QC and were submitted for RNA-sequencing. Libraries were constructed using the NuGEN Trio RNA-Seq kit. Sequencing was completed on the Illumina NovaSeq, with paired-end 2 × 151 bp reads.

For human tissue samples, reads were trimmed with BBDuk v.38.50^61^. Salmon v0.13.1 was used to quantify the transcripts from the RNA-sequence data^62^. Salmon was run in the mapping-based mode, which includes indexing and quantification. The Salmon index was created using the Ensembl release 96 GRCh38 human reference genome with a k-mer setting of 31. Transcript quantification was performed using the ‘–validateMappings’ flag. R was used to extract gene-summarized counts from the salmon quantification output, using the tximport package^63^. Counts were filtered to have an average of more than 3 reads in either the cases or controls. Principal components (PC) plots were generated for each dataset. One sample (CHCO86) was removed from further analyses due to an insufficient mapping rate and not clustering with the other samples in the PC analysis. Counts were normalized using DESeq2^64^.

### Network analysis

Network analysis of *PLG* was performed with NetworkAnalyst 3.0^23–25^. A list of 32 genes previously found to be associated with OM^22^ and the IMEx Interactome generic protein–protein interaction database were used as input. Significant pathways were identified using the databases from the Kyoto Encyclopedia of Genes and Genomes (KEGG), Reactome, Gene Ontology (GO), and PANTHER via the Function Explorer tool in NetworkAnalyst. Functions associated with the network that had an FDR-adjusted *p*-value less than 0.05 were considered significant.

### Microbiome sequencing and analysis

16S rRNA sequencing and microbiome analysis was performed for 42 middle ear and 77 nasopharyngeal samples from Coloradan individuals with OM. To construct the 16S amplicon library, broad-range amplification and sequence analysis of 16S rRNA genes to determine bacterial profiles were used with previously described methods^16^. Analysis of the alpha-diversity indices of species richness, diversity, abundance and evenness (Chao1, Goods coverage, observed species index, Shannon Diversity Index) were tested for significance in association with *PLG* c.1414G > A genotype via t-test. Individual OTU associations were determined using Wilcoxon test for taxa that passed a prevalence threshold of 10% and relative abundance greater than 1%. R software was used for data analyses and generating figures.

### Molecular modeling for rare PLG variants p.Arg261His, p.Ala494Val and p.Ile682Asn

For the three rare variants that we identified, protein models of PLG (PDB ID 4DUU) and the rare variants p.Arg261His, p.Ala494Val and p.Ile682Asn were constructed in Phyre2 and homology was visually analyzed and compared in DeepView/Swiss-PdbViewer^65,66^.

## Data availability

Novel variants were deposited in ClinVar (accession numbers SCV000996504.1, SCV000996506.1, SCV000996507.1, VCV000013583.6 and VCV000692202.1). RNA-seq data from cholesteatoma and middle ear mucosal tissues are available in dbGaP phs001941.v1.p1. Demultiplexed paired-end 16S rRNA sequence data are accessible in the NCBI Short Read Archive under Accession Number PRJNA439435.

## Supplementary information

Supplementary information.
