# Supplementary material for: Multi-omic studies on missense PLG variants in families with otitis media
Source: Sci Rep. 2020 Sep 14;10:15035. doi: 10.1038/s41598-020-70498-w (PMC7490366; doi:10.1038/s41598-020-70498-w)
Supplement: Supplementary file 1 — Supplementary information. [file 41598_2020_70498_MOESM1_ESM.pdf]

Multi-omic Studies on Missense *PLG* Variants in Families with Otitis Media

Tori C. Bootpetch[1], Lena Hafrén[2], Christina L. Elling[1,3], Erin E. Baschal[1], Ani W. Manichaikul[4], Harold S. Pine[5], Wasyl Szeremeta[5], Melissa A. Scholes[1,6], Stephen P. Cass[1], Eric D. Larson[1], Kenny H. Chan[1,6], Rafaqat Ishaq[7], Jeremy D. Prager[1,6], Rehan S. Shaikh[7], Samuel P. Gubbels[1], Ayesha Yousaf[8], University of Washington Center for Mendelian Genomics, Todd M. Wine[1,6], Michael J. Bamshad[9], Patricia J. Yoon[1,6], Herman A. Jenkins[1], Deborah A. Nickerson[9], Sven-Olrik Streubel[1,6], Norman R. Friedman[1,6], Daniel N. Frank[10], Elisabet Einarsdottir[11,12], Juha Kere[11,12,13], Saima Riazuddin[7], Kathleen A. Daly[14], Suzanne M. Leal[15], Allen F. Ryan[16], Petri S. Mattila[2], Zubair M. Ahmed[7], Michele M. Sale[4,17,18], Tasnee Chonmaitree[19], Regie Lyn P. Santos-Cortez[1,20]*

[1]Department of Otolaryngology-Head and Neck Surgery, School of Medicine, University of Colorado Anschutz Medical Campus, Aurora, Colorado, USA
[2]Department of Otorhinolaryngology, Head & Neck Surgery, University of Helsinki and Helsinki University Hospital, Helsinki, Finland
[3]Human Medical Genetics and Genomics Program, University of Colorado Anschutz Medical Campus, Aurora, Colorado, USA
[4]Center for Public Health Genomics, School of Medicine, University of Virginia, Charlottesville, Virginia, USA
[5]Department of Otolaryngology, University of Texas Medical Branch, Galveston, Texas, USA
[6]Department of Pediatric Otolaryngology, Children's Hospital Colorado, Aurora, Colorado, USA
[7]Department of Otorhinolaryngology, Head and Neck Surgery, School of Medicine, University of Maryland, Baltimore, Maryland, USA
[8]Institute of Molecular Biology and Biotechnology, Bahauddin Zakariya University, Multan, Punjab, Pakistan
[9]Department of Genome Sciences, University of Washington, Seattle, Washington, USA
[10]Division of Infectious Diseases, Department of Medicine, School of Medicine, University of Colorado Anschutz Medical Campus, Aurora, Colorado, USA
[11]Folkhälsan Institute of Genetics and Molecular Neurology Research Program, University of Helsinki, Helsinki, Finland
[12]Department of Biosciences and Nutrition, Karolinska Institutet, Huddinge, Sweden
[13]Department of Medical and Molecular Genetics, King's College London, London, UK
[14]Department of Otolaryngology, Head and Neck Surgery, University of Minnesota, Minneapolis, Minnesota, USA
[15]Center for Statistical Genetics, Gertrude H. Sergievsky Center, Taub Institute for Alzheimer's Disease and the Aging Brain, Department of Neurology, Columbia University Medical Center, New York, New York, USA
[16]Division of Otolaryngology, Department of Surgery, UCSD School of Medicine and VA Medical Center, La Jolla, California, USA
[17]Department of Public Health Sciences, School of Medicine, University of Virginia, Charlottesville, Virginia, USA
[18]Department of Biochemistry and Molecular Genetics, School of Medicine, University of Virginia, Charlottesville, Virginia, USA
[19]Department of Pediatrics, Division of Infectious Diseases, University of Texas Medical Branch, Galveston, Texas, USA
[20]Center for Children's Surgery, Children's Hospital Colorado, Aurora, Colorado, USA

Supplementary Table S1. Alpha diversity indices for microbiome samples in the Colorado cohort according to carriage of the *PLG* c.1414G>A (p.Asp472Asn) variant

α-diversity indices	*p-value, all ethnicities*	*p-value, White only*
Middle Ear	*n*=42	*n*=27
• Goods	0.77	0.49
• S_{obs}	0.48	0.46
• Chao1	0.56	0.55
• ShannonH	0.78	0.72
• ShannonE	0.90	0.79
Nasopharynx	*n*=77	*n*=53
• Goods	0.25	0.14
• S_{obs}	0.88	0.40
• Chao1	0.79	0.31
• ShannonH	0.87	0.76
• ShannonE	0.98	0.91

Supplementary Table S2. Characteristics of individuals with otitis media by cohort[1]

Variable	*Texas (n=257)*	*Colorado (n=85)*	*Minnesota (n=140)*	*Helsinki (n=231)*	*Pakistan (n=19)*
Otitis media type					
- %Recurrent/acute	85%	64%	40%	32%	37%
- %Chronic/effusive	14%	27%	8%	10%	42%
- %Both/either[2]	1%	9%	52%	58%	21%
Otitis media surgery					
- %Ventilation tubes	100%	95%	NA	59%[3]	0
- %Tympanoplasty	0	5%	NA	0	0
Median age (years)	1.9	2.0	16	12	17
%Female	45%	31%	49%	43%	38%
%Breastfed	77%	89%	NA	87%	NA
%Smoking exposure	4%	17%	NA	28%	NA
%Ethnicity	66% White, 18% Hispanic, 9% Black, 1% Asian, 7% other	75% White, 10% Hispanic, 3% Asian, 1% Black, 11% other	97% White, 3% other/ mixed	100% White	100% Asian
%(+) Family history	61%	34%	75%	100%	100%
DNA source	Saliva	Saliva	Blood	Blood	Blood
Tests performed	Sanger seq, Fisher exact, logistic regression, TDT	Sanger seq, Fisher exact, logistic regression, TDT, RNA-seq, microbiome	Exome seq, Sanger seq, Fisher exact	Exome seq, Sanger seq, Fisher exact	Exome seq, Sanger seq

[1]Except for the indigenous population and Pakistani families, all individuals and families with otitis media were first identified upon referral for otitis media surgery.

[2]Due to the younger age of affected individuals in Colorado, majority of affected individuals were diagnosed with recurrent acute otitis media. On the other hand, in the Finnish families a larger proportion of individuals were diagnosed with both recurrent acute or chronic effusive otitis media at different time points. In the indigenous Filipino population, although majority of individuals have chronic otitis media due to lack of access to specialist care, 20% had previous diagnoses of either acute or chronic otitis media. These observations support the concept that for a sizeable proportion of our study population, otitis media occurs within a spectrum of disease rather than having a clear delineation between acute/recurrent and chronic/effusive forms of otitis media.

[3]The Finnish cohort includes 217 multi-affected families with exomes from affected individuals and clinical data from all family members with DNA samples.

NA, not available.

Supplementary Table S3: Significant pathways associated with PLG and related genes[1] in network analysis[2]

Reactome	***Pval***	***AdjP***
Dissolution of Fibrin Clot	0.0000201	0.0124
Platelet degranulation	0.0000225	0.0124
Response to elevated platelet cytosolic Ca2+	0.0000265	0.0124
Regulation of Insulin-like Growth Factor (IGF)	0.0000937	0.0328
GO:BP	***Pval***	***AdjP***
Cell substrate adhesion	0.0000913	0.0391
Myoblast differentiation	0.000138	0.0391
Exocytosis	0.000191	0.0391
Leukocyte migration	0.000191	0.0391
Cell activation	0.000272	0.0447

[1]Significant loci identified in previous GWAS studies of otitis media.
[2]Network analysis described in methods.

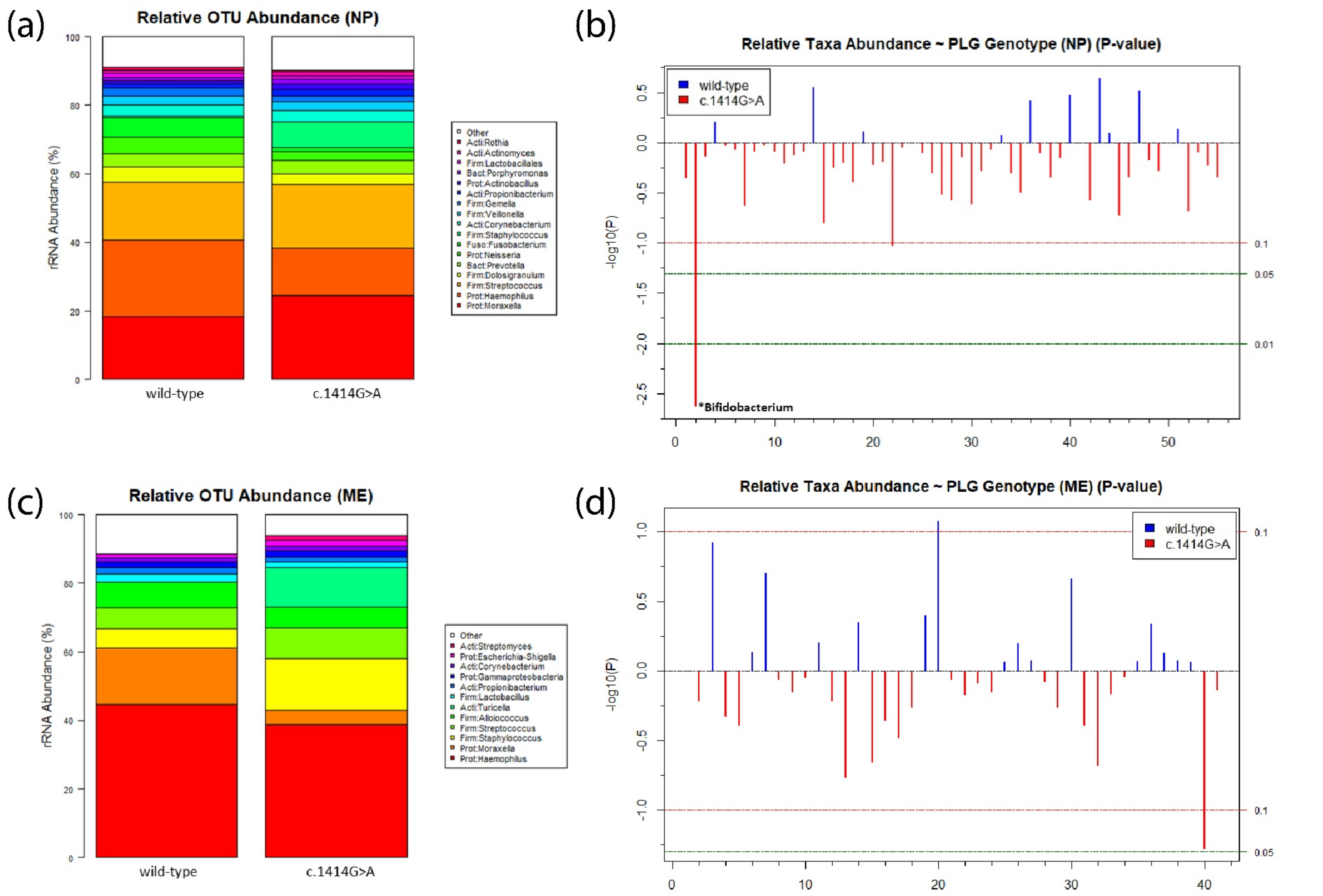

Supplementary Figure S1. Relative abundance of middle ear and nasopharyngeal samples from OM patients.

(a, c) Overall relative taxa abundance (as percent abundance of rRNA relative to total microbiota rRNA). "Other" consists of taxa with overall <5% prevalence and <0.01% abundance. (b, d) Plotted transformed *p*-values of individual taxon (>10% prevalence and >1% relative abundance) associations with genotype (associations with wild-type in blue and associations with c.1414G>A (p.Asp472Asn) variant in red). The only significant association is *Bifidobacterium* with the c.1414G>A (p.Asp472Asn) variant within the NP samples (p=0.0024).

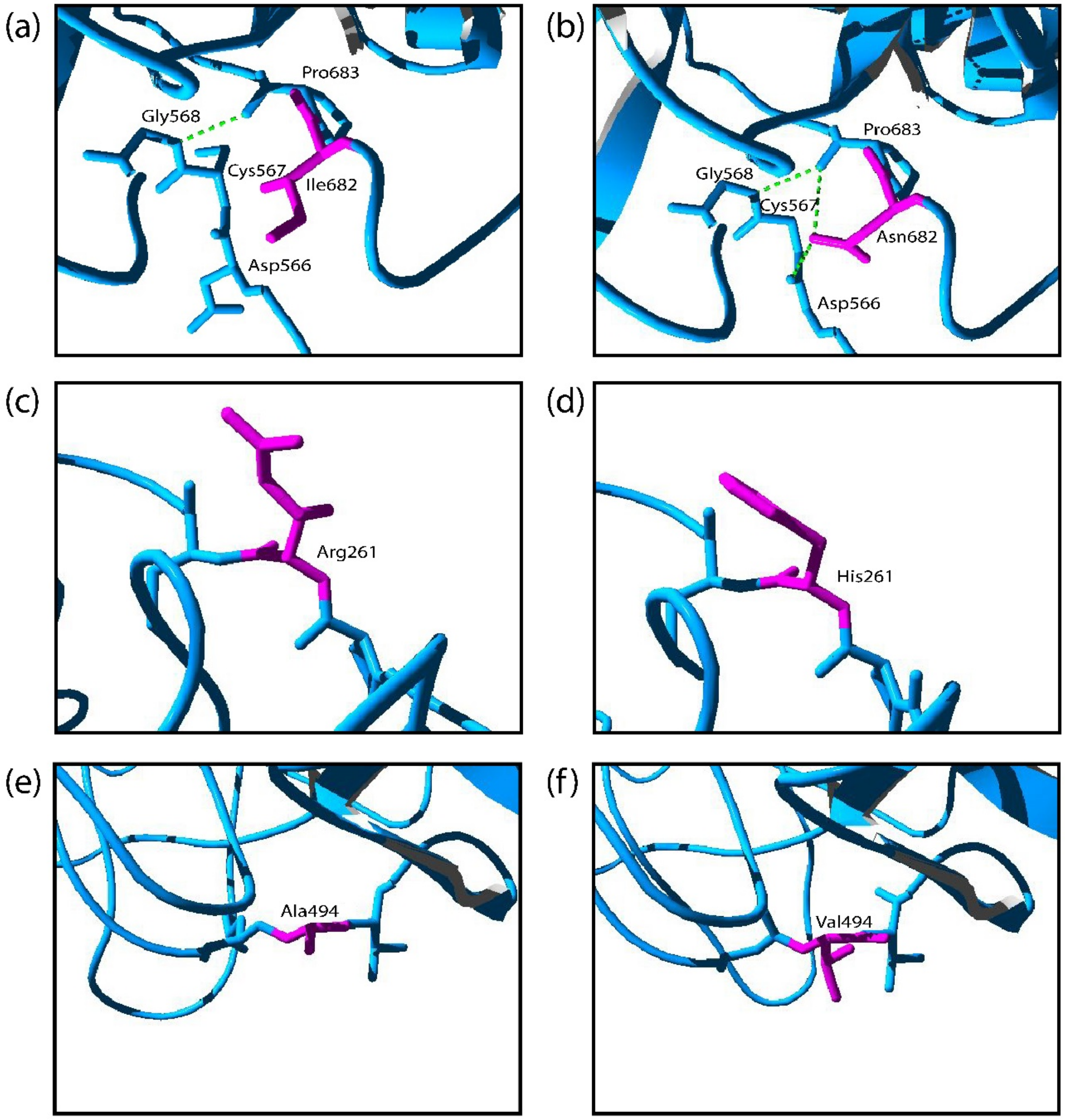

Supplementary Figure S2. Molecular modeling for novel, rare missense PLG variants.

(a) Image of Ile682 showing H-bond with Pro683. (b) Asn682 has two extra H-bonds with Cys567 and Pro683. (c, d) p.Arg261His does not cause any loss or gain of H-bonds but it is predicted to cause structural changes in the interface between kringle domains and binding of chloride ion. (e, f) No obvious differences due to the p.Ala494Val variant.

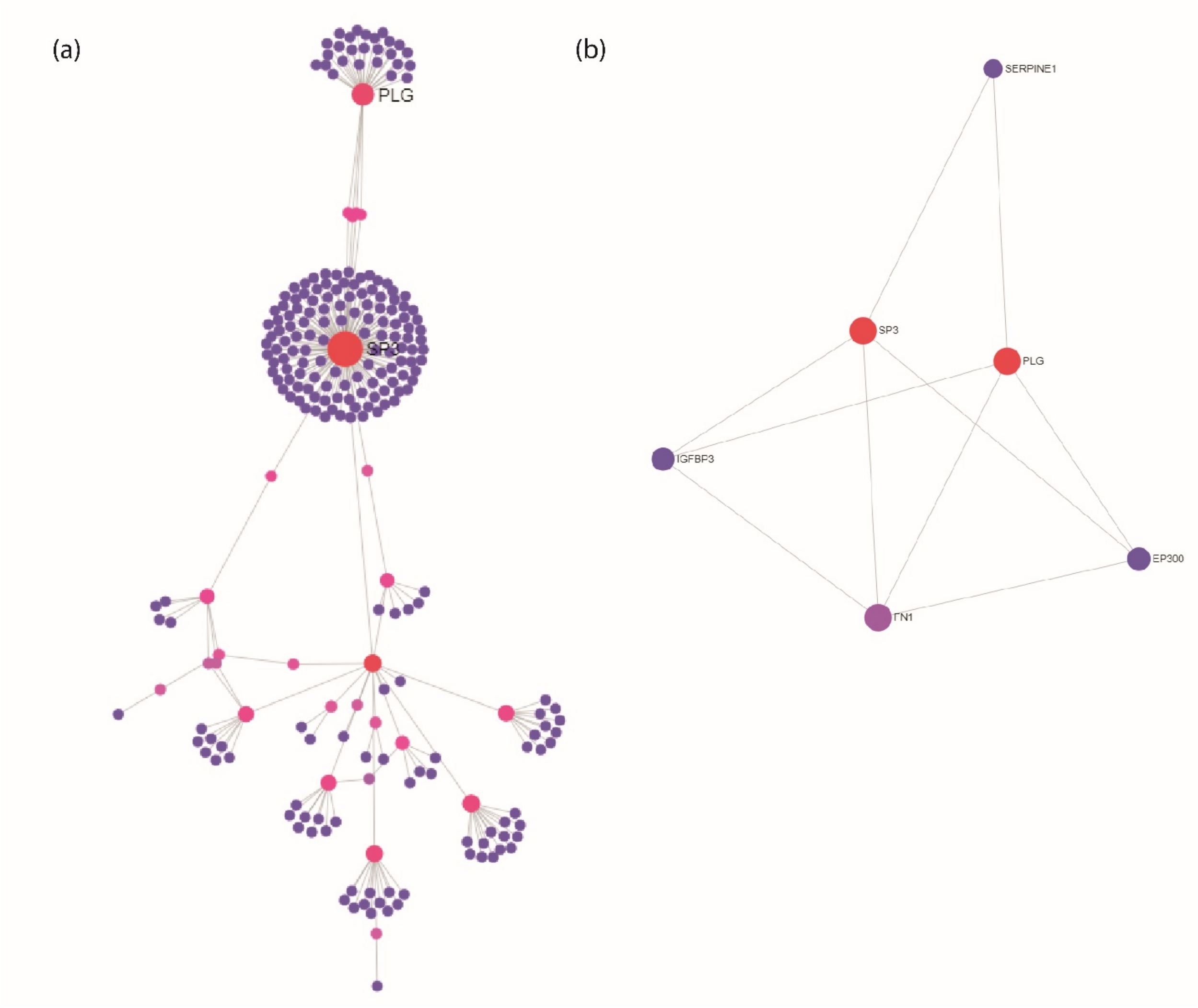

Supplementary Figure S3. Network of otitis media susceptibility genes including *PLG*.

(a) Network connecting *PLG* to other otitis media susceptibility genes identified from previous GWAS. (b) Subnetwork shows that *PLG* is connected to *EP300*, *FN1*, *IGFBP3* and *SERPINE3*, four genes through which *PLG* is connected to another OM susceptibility candidate gene from GWAS, *SP3*.
